# Differential alterations in peripheral tryptophan pathways in methamphetamine versus MDMA users are linked to their contrasting psychiatric symptoms

**DOI:** 10.1038/s41398-026-04069-4

**Published:** 2026-05-21

**Authors:** Francesco Bavato, Andrea Steuer, Anna M. Jacobsen, Amelie Zacher, Josua Zimmermann, David M. Cole, Antje Opitz, Markus R. Baumgartner, Ann-Kathrin Stock, Christian Beste, Boris B. Quednow

**Affiliations:** 1 Experimental Pharmacopsychology and Psychological Addiction Research, Department of Adult Psychiatry and Psychotherapy, University Hospital of Psychiatry Zurich, University of Zurich, Zurich, Switzerland; 2 Center of Functionally Integrative Neuroscience, Department of Clinical Medicine, Aarhus University, Aarhus, Denmark; 3 Institute of Forensic Medicine, University of Zurich, Zurich, Switzerland; 4 Neuroscience Center Zurich, Joint Center of University of Zurich and Swiss Federal Institute of Technology Zurich, Zurich, Switzerland; 5 Translational Psychiatry Lab, University Psychiatric Clinics Basel, University of Basel, Basel, Switzerland; 6 Department of Child and Adolescent Psychiatry, TU Dresden, Dresden, Germany

**Keywords:** Diagnostic markers, Physiology

## Abstract

Methamphetamine (METH, “Crystal Meth”) and 3,4-methylenedioxymethamphetamine (MDMA, “Ecstasy”) are two types of substituted amphetamines that share structural-chemical similarities but exhibit contrasting acute and chronic effects including addictive liability. Tryptophan (TRY) pathways are involved in pleiotropic physiological functions at the interface of brain-body connections. Preclinical evidence suggests that amphetamines may modulate these pathways and, thus, indirectly influence brain functions via persistent alterations of peripheral metabolites. However, little is known about alterations of TRY-related metabolites in the blood and their clinical implications in chronic users of MDMA and METH. Hence, we characterized serum levels of TRY-related metabolites in a comparative cross-sectional study including *n* = 36 chronic MDMA users, *n* = 33 chronic METH users, and *n* = 71 sex-matched, healthy controls. An ultra–high performance liquid chromatography–mass spectrometry method was used to determine TRY metabolites. Combining metabolite levels, metabolic ratios, and network analysis we found robust evidence of divergent pathway alterations between METH and MDMA users. Chronic METH use was particularly associated with a depletion of serum TRY and serotonin levels, and a general activation of kynurenine pathways, while chronic MDMA use was linked to a selective activation of the OH-kynurenine metabolic branch. Metabolite changes were associated with the severity of psychopathology in the depression and psychosis domains across groups. Altogether, our findings demonstrate differential changes of serum TRY pathways in chronic MDMA and METH users. Persistent alterations of these pathways might contribute to the contrasting clinical profile of the substances and constitute a peripheral dimension of neurochemical plasticity with relevant implications for therapeutic targets.

## Introduction

Methamphetamine (METH, “Crystal Meth”) and 3,4-methylenedioxymethamphetamine (MDMA, “Ecstasy”, “Molly”) are two types of substituted amphetamines that are widely used for recreational purposes in Europe and globally [1]. Despite their structural similarity and partially overlapping mechanisms of action, METH and MDMA differ markedly in their acute and long-term effects, as well as in their addictive liability. Both substances act as strong monoamine releasers, but they differ in their affinity for specific monoamine transporters: METH primarily promotes the release of dopamine (DA) and noradrenaline (NE), whereas MDMA predominantly releases serotonin (5-HT) and NE [2, 3]. Chronic use of METH and MDMA induces neuroplastic adaptations in monoaminergic systems. METH has been associated with alterations in DA, NE, and 5-HT circuits, while MDMA has been more selectively linked to changes in serotonergic pathways, although with some species-dependent differences [4–7]. Putative neurotoxic effects and consequent structural brain alterations have been widely reported in both chronic METH users and MDMA users [8–10].

Clinically, chronic METH use is associated with affective symptoms (e.g., depressive symptoms and suicidal behaviour), psychotic-like experiences (e.g., paranoia, hallucinations, delirium, and delusion) increased impulsivity and violent behaviour, as well as high liability to addiction [3]. Consequences of chronic METH use also include dysfunctions in cognitive performance, decision-making, and empathy [11, 12]. Notably, chronic METH use is also associated with relevant physical harm such as cardiovascular pathology (e.g., hypertension and myocardial pathology), reduced liver and kidney function, skin lesions (e.g., excoriations, ulcers, cellulitis), and poor oral hygiene (e.g. damaged and discoloured teeth, also called “meth mouth”) [3]. In contrast, chronic MDMA use is associated with a low risk of addiction and has limited relevance in clinical psychiatry, apart from transient depressive symptoms that often emerge a few days after use (a phenomenon referred to as “midweek blues”) [13]. However, whether chronic MDMA use can elevate the long-term risk for psychiatric disorders remains a matter of debate [14]. Like METH, chronic MDMA use has also been consistently associated with long-term cognitive deficits, although with higher domain-specificity manifesting as impairments in long-term memory and executive function, while socio-cognitive functions are usually preserved [15, 16]. Despite decades of research, the mechanistic basis for the markedly different clinical outcomes of chronic METH and MDMA use remains insufficiently understood, particularly given their overlapping pharmacological profiles.

Peripheral 5-HT and tryptophan (TRY) pathways are involved in pleiotropic physiological functions at the interface of brain-body connections. Blood 5-HT levels have been shown to modulate sensory neuron activity and interoceptive signaling independently of 5-HT neurotransmission in the brain [17]. In this context, a reduction of blood 5-HT was causally linked to fatigue and cognitive impairments in the post-acute phase of viral infections. The 5-HT precursor TRY is an essential amino acid obtained exclusively through diet, critical for both multi-organ protein synthesis and brain-specific 5-HT metabolism through availability-limiting mechanisms [18]. Experimental TRY depletion in humans has been associated with depressive symptoms, memory impairment and increased aggression [19]. Circulating TRY catabolites produced in the liver and in immune cells (Kynurenine [KYN] metabolites) [20] or by gut microbiota (indole [IND] metabolites) [21] can cross the blood-brain barrier (BBB) and exert psychoactive functions by modulating glutamatergic transmission (via N-methyl-D-aspartate [NMDA] and α7 nicotinic acetylcholine receptors), microglial activation, and mRNA expression (via aryl hydrocarbon receptor signalling) [20]. In particular, the balance between kynurenic acid (KYNA) and hydroxy-kynurenine (OH-KYN), which are alternative products of KYN metabolism, has been found to shape neural-glial crosstalk; however, only OH-KYN and KYN can directly cross the blood brain barrier, while KYNA is produced from KYN in the astrocytes [22].

**Fig. 1:**
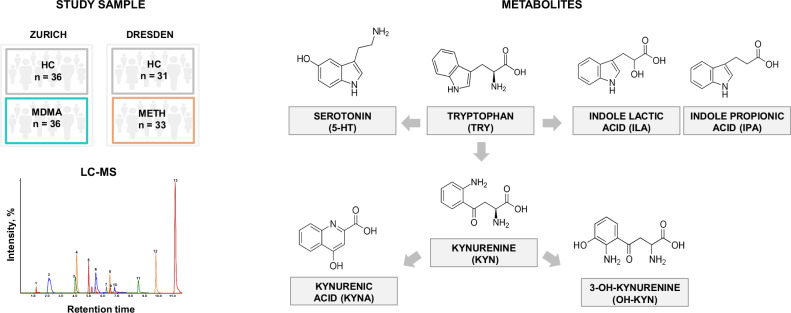
Study overview and analytical workflow. Upper left: group composition showing chronic MDMA users and their matched healthy controls (HC), and chronic METH users and their matched HC. Lower left: representative liquid chromatography–mass spectrometry (LC–MS) chromatogram illustrating metabolite detection (signal intensity over retention time). Right: schematic overview of the tryptophan metabolic pathway, including the serotonin, kynurenine, and indole branches with the quantified metabolites.

Preclinical evidence indicates that substituted amphetamines may modulate TRY-related metabolic pathways [23] and, thus, indirectly influence brain functions via body-first neurochemical changes. However, in humans, little is known about how chronic MDMA and METH use affects blood levels of these metabolites, or how such peripheral alterations relate to clinical symptoms. Identifying whether METH and MDMA differentially affect TRY pathways could clarify the biological mechanisms underlying substance-related psychopathology and reveal potential targets for novel therapeutic interventions. Notably, Wang et al. previously reported reduced TRY levels, increased KYN/TRY ratios, and activation of the OH-KYN branch in individuals with METH use [24]. TRY depletion has also been described in METH users with comorbid HIV infection [25, 26]. However, existing human studies have been limited by small sample sizes [26], comorbid medical conditions (e.g., HIV) [27], lacking investigation of KYN and IND metabolites [25–28], as well as limited documentation of substance use patterns and psychiatric symptomatology [25–28]. Moreover, previous studies only focused on METH with little to no investigation on MDMA, thereby precluding a clear understanding of the specific links between substance use, neurochemical alterations, and clinical outcomes. Therefore, we aimed at characterizing serum levels of 5-HT, TRY, indole lactic acid (ILA) and indole propionic acid (IPA), KYN, KYNA, and OH-KYN in chronic METH and MDMA users (see Fig. 1). We hypothesized that chronic use of METH and MDMA would be associated with distinct alterations in serum concentrations of these metabolites compared to controls and relative to each other, reflecting differences in both pharmacology and patterns of use. We also conducted an exploratory network analysis to compare the organization of the metabolic pathway between MDMA and METH users in terms of edge weights, network centrality and eccentricity. Finally, we hypothesized that the alteration of peripheral metabolite levels would predict the distinct psychopathological profiles associated with the chronic use of each substance.

## Methods

### Participants

A total of *N* = 203 participants were initially recruited between June 2019 and July 2021 by means of online media and flyer advertisements placed in institutions involved in substance use information and prevention, addiction clinics, and in public places. The final sample considered for this analysis included 36 chronic MDMA users and 36 age-, sex-, education-, verbal intelligence-, and nicotine use-matched healthy controls (HC) from Zurich, Switzerland, as well as 33 chronic METH users and 35 age- and sex-matched HC from Dresden, Germany. Exclusions from current analysis were based on lack of availability for blood samples (*n* = 27), not fulfilling the requirements from substance use criteria according to toxicological data (*n* = 31), relevant medical conditions (*n* = 4), or pregnancy (*n* = 1), according to previous publications [11].

All participants were aged 18 to 45 years. Substance users were eligible with a minimum of 25 lifetime occasions of MDMA or METH use and recent use within the last 12 months. Either MDMA or METH had to be the primary illicit substance for the respective group. General exclusion criteria included i) pregnancy or breastfeeding, ii) acute or previous severe somatic or neurological disorders, iii) severe DSM-IV Axis I psychiatric disorders such as autism and schizophrenia, iv) current intake of psychotropic medication (not applicable in the METH user group) and v) daily cannabis use (not applicable in the METH user group). Controls were excluded if they reported illicit substance use on more than 15 lifetime occasions (except cannabis) or intake of stimulants in the past 4 months (confirmed by hair analysis). All participants were asked to abstain from illicit substances for at least 48 h (verified through urine substance screening) and alcohol for 24 h before testing. Daily cannabis use and psychotropic medication were not exclusion criteria in the METH group due to their high prevalence and the risk of substantially reducing sample size and introducing a strong selection bias. The influence of cannabis and psychotropic medication use was therefore investigated in sensitivity analyses.

The study was approved by the Ethics Committees of the Canton Zurich (BASEC-Nr. 2018–02125) and the TU Dresden (EK 69022018). All participants provided written informed consent and received compensation for their participation in accordance with the Declaration of Helsinki. Data on social cognition (whole sample) and brain imaging (MDMA sample only) from the same dataset have been published before [11, 29, 30].

### Clinical and substance use assessment

All participants were screened for common DSM-IV Axis I disorders by trained examiners. Substance use disorders were additionally assessed with the Structured Clinical Interview for DSM-5 Axis I disorders. Subjective substance use history was further assessed with the standardized and structured Interview for Psychotropic Drug Consumption [31]. To objectively characterize substance use during the four months prior to testing, proximal hair samples of 4 cm were taken from the occiput. If a participants’ scalp hair length was not sufficient, body hair was sampled. Liquid chromatography-tandem mass spectrometry (LC-MS/MS) was used for toxicological hair analysis [32]. To additionally screen for recent illicit substance intake, multi-substance urine tests were conducted prior to the tasks. Self-report screening questionnaires were used to evaluate (i) symptoms of depression using the Center for Epidemiologic Studies Depression Scale (CESD) [33], (ii) attention-deficit/hyperactivity disorder (ADHD) with the ADHD Self-Rating Scale (ADHD-SR) [34], (iii) psychotic-like experiences with the Community Assessment of Psychic Experience (CAPE) [35]; and (iv) subjective trait impulsivity with the Barratt Impulsiveness Scale (BIS-11) [36]. To estimate the premorbid verbal intelligence (verbal IQ), the standardized German vocabulary test (Mehrfachwahl-Wortschatz-Intelligenztest) was applied [37].

### Assessment of blood markers

Blood serum samples were drawn using silica and gel containing tubes (BD Vacutainer). All samples were collected in the afternoon (14-16:00) after a light meal. Samples were centrifuged for 15 min at 2000 rpm and subsequently stored at <−20 °C. Metabolite levels were analysed using an ultra–high performance liquid chromatography system (Thermo Fisher, San Jose, CA, USA), coupled to a 5500 linear ion trap quadrupole mass spectrometer (Sciex, Darmstadt, Germany), according to a well-established procedure from the same lab [38]. The analyst was blinded to group allocation.

### Statistical analysis

#### Pre-processing and sample characteristics

Statistical analyses were conducted in Python (v3.9.6). Demographic and substance use data were analysed using Pearson’s chi-square tests and analyses of variance (*scipy* v1.16.0). All metabolite levels were winsorized in order to account for outliers that fell outside two interquartile ranges from the median (*pandas* v2.3.1). Metabolite levels were z-transformed per site using means and standard deviations (SD) of the respective HC sample to account for potential site-related differences in sex and diet [39, 40]. This approach generates direct comparisons between each user group and its respective matched HC in the calculation of average z-scores. For the following analyses HC were pooled and both users groups included in the same models to allow direct MDMA–METH comparisons and to enable more conservative and robust adjustment for covariates across the whole sample. Metabolic ratios were calculated to further quantify the general activation of the KYN pathway (KYN/TRY), of the serotonin pathway (5-HT/TRY), the alternative activation of the OH-KYN (OH-KYN/TRY) and KYNA (KYNA/TRY) branches and their relative balance (KYNA/OH-KYN) according to previous publications [41].

#### Group comparisons

Group differences were assessed via linear mixed-effects model (LME; *statsmodels* v0.14.5) that predicted z-standardized metabolite levels or ratios with group and sex as fixed factors, random intercepts modelled for participants, and age and body mass index (BMI) as covariates. Sex, age and BMI were included considering their influence on TRY catabolites and 5-HT levels [39]. Pairwise comparisons for metabolites and ratios showing significant group differences were then assessed with Mann-Whitney U tests (to account for non-normal distribution and variance heterogeneity). Power analysis indicated that, with α = 0.05 and power (1−β) = 0.80, moderate group effects (f = 0.26) could be detected in an ANOVA model with 3 groups. Multiple-comparison correction was performed using false discovery rate (FDR) method. Effect size Cohen’s d was reported for group differences based on means and SDs. A sensitivity analysis additionally included urine toxicology results in the LME (METH users: amphetamine *n* = 6, THC *n* = 5, methamphetamine *n* = 10, any psychoactive substance *n* = 12; no urine positivity in MDMA users or controls) as a categorical predictor to control for potential acute substance effects. Additional separate LMEs included smoking (cigarettes/week), alcohol use (grams/week), cocaine use (total grams in 12 months), and current psychotropic medication intake (yes/no) as covariates for control analyses.

#### Dose-response relationships

Dose-response relationships between cumulative substance use over the past 12 months and alterations in metabolite levels and metabolite ratios were investigated using LMEs corrected for sex, age and BMI in METH and MDMA users separately. Only metabolites and ratios showing significant group differences compared to HC were included in these analyses. Hair data were reported descriptively but were not included in further statistical analyses due to limited sample availability from the Dresden site.

#### Network analyses

To investigate further potential alterations in the organization of the metabolic pathways, explorative network analyses were performed using NetworkX (v3.5) [42]. First, correlation matrices across all z-standardized metabolites were calculated based on Spearman’s rank correlation coefficients for each group. Then the difference between correlation matrices for METH or MDMA users and their respective HC groups was calculated. These values were used as network edges, with each metabolite acting as a node. The following network parameters were computed: betweenness centrality, closeness centrality, eigenvector centrality, and eccentricity. This approach captures differential metabolic connections across groups by directly comparing all possible relationships between metabolites (MDMA vs. HC and METH vs. HC) and quantifying how they change across samples. Moreover, a paired t-test was performed to compare edges values across metabolites between separate networks (MDMA-HC vs. METH-HC).

#### Psychopathology associations

Potential associations between metabolite levels, ratios, and psychopathology scores were investigated using LME models adjusted for age, sex, and BMI. These were examined across groups to reflect a dimensional approach, capturing the full range of symptom severity while increasing statistical power and variance relative to within-group analyses. FDR correction was used to control for multiple comparisons for each metabolite.

## Results

### Demographic and clinical characteristics

Demographic, clinical, and substance use characteristics are summarized in Table 1. Among demographic and clinical characteristics, significant group differences were identified in sex distribution, years of education, as well as ADHD-SR, CESD, BIS, and CAPE scores. Regarding alcohol, nicotine, and cannabis use, significant group differences were observed in self-reported consumption frequency, duration, and dose, with highest use in METH users. Substance use in HC was negligible and in line with inclusion and exclusion criteria.

**Table 1:** Demographic and clinical characteristics.

	HC, *n* = 71	MDMA, *n* = 36	METH, *n* = 33	*MDMA vs METH* *Statistic*	*MDMA vs METH* *P-value*
Sex, female/male	30 / 41	20 / 16	8 / 25	*χ*^*2*^ = 6.99	**0.036**
Age, years	30.03 (6.5)	30.25 (7.0)	29.82 (6.29)	F = 0.036	0.96
BMI	23.7 (3.89)^**#**^	23.56 (4.16)	25.4 (4.10)	F = 3.66	**0.028**
Education, years	10.23 (1.19)^**#**^	10.31 (1.47)	9.64 (0.98)	F = 3.17	**0.045**
Verbal IQ, score	104.63 (9.19)	102.0 (10.67)	99.70 (9.58)	F = 3.02	0.052
ADHD-SR, score	10.94 (7.59)**	13.14 (10.11)	16.63 (10.36)	F = 4.42	**0.014**
CESD, score	9.23 (7.90)***^#^	12.25 (11.28)	20.33 (13.84)	F = 12.34	**<0.001**
BIS, score	61.6 (8.35)***	65.06 (10.35)	69.42 (9.41)	F = 8.15	**<0.001**
CAPE, score	1.35 (0.19)**^#^	1.41 (0.31)	1.60 (0.40)	F = 8.47	**<0.001**
Smoking y/n	23 / 48**^###^	19 / 17	31 / 2	*χ*^*2*^ = 34.2	**<0.001**
Cigarettes/week	37 (36)**^###^	53 (49)	92 (49)	F = 10.42	**<0.001**
Alcohol y/n	69 / 2	36 / 0	33 / 0	*χ*^*2*^ = 1.97	0.37
Alcohol gram/week	74.29 (111.5)	142.16 (182.78)	121.60 (222.51)	F = 2.30	0.10
Cannabis use y/n	43 / 28***	32 / 4	33 / 0	*χ*^*2*^ = 23.66	**<0.001**
Cannabis, gram/week	0.02 (0.09)***^##^	0.20 (0.44)	1.81 (3.04)	F = 16.76	**<0.001**
Cocaine, y/n	7 / 64***	26 / 10	27 / 6	*χ*^*2*^ = 64.7	**<0.001**
Cocaine, grams in last 12 months	0.005 (0.02)*	3.16 (8.31)	0.36 (0.97)	F = 6.82	**0.0015**
Cocaine, hair concentration, pg/mg	1.84 (16.85)***	247.92 (445.14)	51.38 (145.57)	F = 11.19	**<0.001**
METH, y/n	0 / 71***^###^	0 / 36	33 / 0	*χ*^*2*^ = 140	**<0.001**
METH, grams in last 12 months	0 (0)^**###**^	0 (0)	90.4 (108.0)	F = 50.21	**<0.001**
METH, hair concentration, pg/mg	0 (0)**^###^	36.58 (213.56)	9592.57 (13021.64)	F = 26.65	**<0.001**
MDMA, y/n	2 / 69***	36 / 0	27 / 6	*χ*^*2*^ = 44.31	**<0.001**
MDMA, grams in last 12 months	0 (0)***^###^	6.14 (6.1)	0.18 (0.54)	F = 50.21	**<0.001**
MDMA, hair concentration, pg/mg	0 (0)**^#^	3362.81 (6560.78)	75.33 (145.80)	F = 10.89	**<0.001**
Positive urine tests (any drug) y/n	0 / 71***^###^	0 / 36	12 / 21		**<0.001**

Values reflect mean and standard deviations (in brackets). Statistical tests: ANOVA (all groups) for quantitative data and chi-squared test or fisher test (all groups) for frequency data; significant p-values are shown in bold. Post-hoc tests: Users vs. healthy controls (HC): *p < 0.05, **p < 0.01, ***p < 0.001; MDMA vs. METH users: #*p* < 0.05, ##*p* < 0.01, ###*p* < 0.001.

Some data presented in this table have been reported previously [11].

*IQ* intelligence quotient, *ADHD-SR* ADHD Self-Rating Scale, *CESD* Center for Epidemiologic Studies Depression Scale, *BIS* Barratt Impulsiveness Scale, *CAPE* Community Assessment of Psychic Experience, *BMI* Body Mass Index.

### Group differences in serum metabolite levels and metabolite ratios

**Fig. 2:**
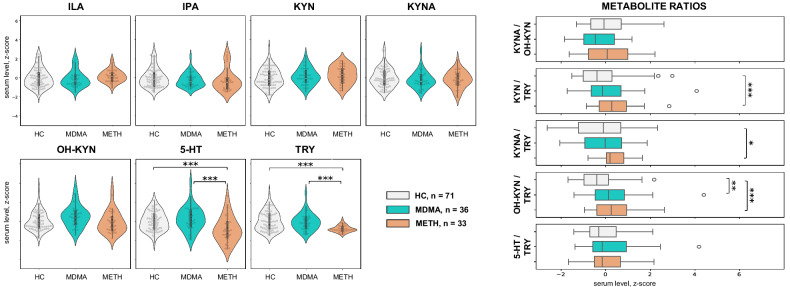
Group differences in metabolite levels. Left panel: violin plots with individual jitters showing metabolite levels (z-transformed) across samples. Right panel: boxplots showing ratios for metabolite levels across samples. Abbreviations: HC: Healthy Controls; ILA: indole lactic acid; IPA: indole propionic acid; KYN: Kynurenine; KYNA: kynurenic acid; OH-KYN: hydroxy-kynurenine; 5-HT: serotonin; TRY: tryptophan. *p < 0.05, **p < 0.01, ***p < 0.001.

Serum metabolite levels and ratios across groups are reported in Fig. 2. When adjusted for participant age, sex, and BMI using LMEs, group effects were found for 5-HT levels (*p* = 0.01, d = 0.37; pairwise comparisons FDR corrected: MDMA vs. METH: U = 316, *p* = 0.001, d = 0.53; MDMA vs. HC: U = 1128, *p* = 0.32, d = 0.22; METH vs. HC: U = 1683, *p* = 0.001, d = 0.78) and TRY levels (*p* < 0.001, d = 0.64; pairwise comparisons FDR corrected: MDMA vs. METH: U = 253, *p* < 0.001, d = 1.05; MDMA vs HC: U = 1321, *p* = 0.78, d = 0.07 ; METH vs. HC: U = 1749, *p* < 0.001, d = 1.05), demonstrating TRY and 5-HT reduction in METH users. LMEs also demonstrated KYN pathway activation in METH users (KYN/TRY, *p* = 0.001, d = 0.49; pairwise comparisons FDR corrected: MDMA vs. METH: U = 708, *p* = 0.26, d = 0.38 ; MDMA vs. HC: U = 1002, *p* = 0.10, d = 0.35; METH vs. HC: U = 656, *p* < 0.001, d = 0.71), KYNA branch activation in METH users (KYNA/TRY, *p* = 0.018, d = 0.35; pairwise comparisons FDR corrected: MDMA vs. METH: U = 760, *p* = 0.07, d = 0.58; MDMA vs. HC: U = 1246, *p* = 0.83, d = 0.02; METH vs. HC: U = 819, *p* = 0.04, d = 0.52), and OH-KYN branch activation in both METH and MDMA users (OH-KYN/TRY, *p* < 0.001, d = 0.57; pairwise comparison FDR corrected: MDMA vs. METH: U = 617, *p* = 0.78, d = 0.04; MDMA vs HC: U = 865, *p* = 0.01, d = 0.60; METH vs. HC: U = 721, *p* = 0.005, d = 0.68).

#### Control analyses for separate study sites

Control analyses using separate group comparisons of MDMA and METH with their respective HC groups confirmed all reported group effects (all *p* < 0.05). Additionally, MDMA users showed higher OH-KYN (U = 463, *p* = 0.037, d = 0.48) and lower KYNA/OH-KYN ratio compared to HC (U = 853, *p* = 0.011, d = 0.678) consistently with the observed OH-KYN branch activation in MDMA users.

### Sensitivity analysis for serum metabolite levels

#### Urine positivity

To clarify if the detected group differences (i.e., 5-HT, TRY, KYN/TRY, KYNA/TRY, and OH-KYN/TRY) are potentially dependent on acute or post-acute effects related to recent substance intake, we included urine positivity to any drug (urine positive cases in HC: *n* = 0; MDMA users: *n* = 0; METH users: *n* = 12) as a factor in the LMEs. Urine positivity did not predict any of these values and ratios (all *p* > 0.05). When considering urine positivity for METH specifically (METH users, *n* = 10), a significant effect was found for 5-HT (positive vs. negative users, mean z-score ± SD: −1.50 ± 0.53 vs. −0.60 ± 1.51; *p* = 0.005, d = 1.42) but not for other values or ratios (all *p* > 0.05). Adding urine positivity for METH in the LME, weakened the group effect for 5-HT levels (*p* = 0.22, d = 0.20), suggesting this alteration to be rather acute/post-acute than chronic/persistent.

#### Other substances and psychotropic medications

In additional sensitivity analyses, alcohol use (grams per week over the last 12 months), nicotine use (cigarettes per week), cannabis use (grams per week over the last 12 months), cocaine use (total grams consumed over the last 12 months), and self-reported use of any psychotropic medication (*n* = 6) were not significantly associated with 5-HT, TRY values or KYN/TRY, KYNA/TRY, or OH-KYN/TRY ratios in the LMEs (all *p* > 0.05). All detected group effects for metabolites and ratios also remained significant after including nicotine, cannabis, cocaine, or psychotropic medication use in the LMEs (all *p* < 0.05), with the exception of 5-HT, which lost the group effect significance after correction for cannabis use, despite persistence of small effect size in the METH vs. HC comparison (*p* = 0.11, d = 0.29). Finally, excluding two HC with history of MDMA use in the models did not change any group comparison.

### Exploratory network analysis

**Fig. 3:**
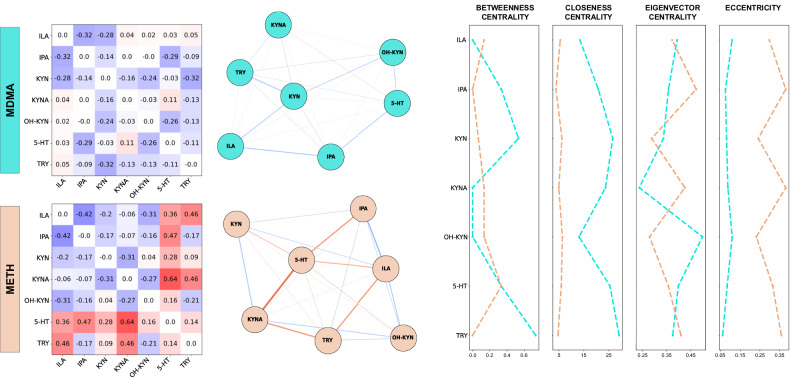
Network analysis of metabolite levels. Left panel: correlation matrices across all z-standardized are calculated based on Spearman’s rank correlation as the difference between correlation coefficients between METH or MDMA users and healthy controls. Central panel: network visualization with metabolites as nodes and correlation coefficients as edges. Right panel: network parameters across nodes separated for each substance. Abbreviations: ILA: indole lactic acid; IPA: indole propionic acid; KYN: Kynurenine; KYNA: kynurenic acid; OH-KYN: hydroxy-kynurenine; 5-HT: serotonin; TRY: tryptophan.

Exploratory network analyses of metabolic pathways are presented in Fig. 3. Here, stronger alterations in network organization could be seen in the METH sample. In the edge matrix, mostly negligible to weak edge weight (correlation coefficient) contrasts (ρ = 0.0–0.3) were found for the MDMA sample, while some moderate to strong contrasts (ρ = 0.3–0.6) are observable in the METH sample (METH vs. HC). A global network estimation also visually confirmed significant differences in the metabolic pathways between MDMA and METH, with stronger alterations in edge weights in the METH sample (t = 2.81, *p* = 0.006). The most consistent differences between the two groups emerged in closeness centrality, which quantifies a node’s communication efficiency, and eccentricity, which quantifies distance to other nodes. Closeness centrality was higher across the network in the MDMA sample, while eccentricity was lower across the network in the MDMA sample than the METH sample.

### Associations between blood markers and 12-month substance use variables

**Fig. 4:**
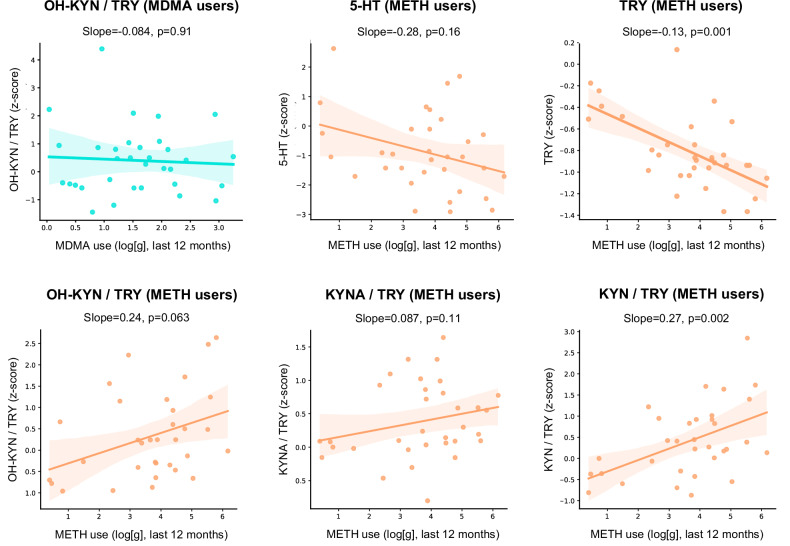
Associations between metabolite levels and substance use. Scatterplots for dose-response relationships between metabolite levels (selected based on significant group effects) and self-reported substance use in the last 12 month. Mixed linear models adjusted for age, sex, and BMI were used. Abbreviations: KYN: Kynurenine; KYNA: kynurenic acid; OH-KYN: hydroxy-kynurenine; 5-HT: serotonin; TRY: tryptophan.

To address potential dose-response relationships, LMEs adjusted for age, sex, and BMI were used to detect associations between metabolites, ratios and self-reported METH or MDMA use in the last 12 months (see Fig. 4). In the METH sample, a negative association was observed with TRY levels, while positive associations were found for the KYN/TRY ratio.

### Associations between blood markers and psychopathology scores

**Fig. 5:**
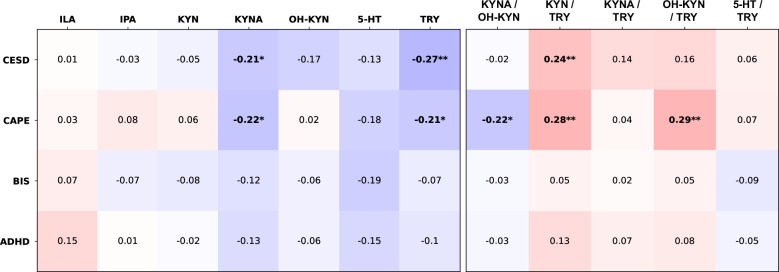
Associations between metabolite levels and psychopathology. Heatmap reporting standardized beta values from mixed linear models adjusted for age, sex, and Body Mass Index (BMI). False Discovery Rate correction was used to control for multiple comparisons for each metabolite. CESD: Center for Epidemiologic Studies Depression Scale; CAPE: Community Assessment of Psychic Experiences; BIS: Barratt Impulsiveness Scale; ADHD: Attention-Deficit/Hyperactivity Disorder; ILA: indole lactic acid; IPA: indole propionic acid; KYN: kynurenine; KYNA: kynurenic acid; OH-KYN: 3-hydroxykynurenine; TRY: tryptophan; 5-HT: serotonin.*p < 0.05, **p < 0.01.

To address potential associations of peripheral metabolites with severity in main self-reported psychopathology scores across the whole sample, LME corrected for age, sex, and BMI were performed (Fig. 5). Significant associations (FDR corrected) were found between KYNA levels, CESD and CAPE scores. TRY levels were also associated with CESD and CAPE scores. Considering metabolite ratios, significant associations were found for KYN balance (KYNA / OH-KYN) with CAPE score, KYN activation (KYN / TRY) with CAPE and CESD scores, and OH-KYN branch activation and CAPE scores.

## Discussion

The present study compared peripheral TRY pathways between chronic METH users, chronic MDMA users, and healthy controls. We provided robust cross-sectional evidence of differential alterations in metabolite concentrations and pathway organization across samples. In particular, METH users showed a reduction of TRY and 5-HT, a general activation of the KYN pathway, and moderate to strong metabolite network changes. On the other hand, MDMA was associated with only small alterations in metabolite balance ratio and no change in absolute values. Metabolite levels and ratios were associated with severity scores in psychiatric symptoms in the total sample suggesting an involvement of peripheral TRY pathways in the differential clinical presentation of chronic use of METH vs. MDMA.

### Chronic METH use is associated with serum TRY reduction

A depletion of blood TRY levels in METH users has been previously suggested, although studies to date employed only limited characterization of substance use variables (no toxicological data) and were highly heterogeneous in term of assays and matrices used [24–28]. Considering that TRY is exclusively acquired with diet, METH-related TRY reduction could either result from decreased input (i.e., dietary lack and malnutrition) or increased metabolism (i.e., conversion into the KYN pathway). Although BMI did not differ substantially between groups, it may not adequately reflect nutritional quality or irregular food intake, and the absence of detailed dietary assessments limits our ability to fully exclude subtle malnutrition-related effects on TRY levels. Based on experimental evidence from preclinical studies (where substance-related malnutrition can be excluded), increased conversion to KYN is seen as the most probable cause [43]. This is also supported by the observed increase in KYN/TRY in our sample. Moreover, the association between METH use in the previous 12 months and both TRY and KYN/TRY in our study, support the suggested dose-response relationship of these effects. Importantly, although elevations in KYN/TRY, KYNA/TRY, and OH-KYN/TRY ratios are most likely driven by TRY reduction, a simple depletion of TRY without downstream pathway activation would be expected to result in proportional reductions in KYN metabolites, which was not observed. Considering the pleiotropic role of TRY, METH-related depletion might have a negative impact on protein synthesis across the body and also limit TRY-dependent 5-HT production in the brain. Notably, TRY pre-treatment in rats administered with METH was found to prevent METH-related behavioural changes [44]. On the other hand, experimental TRY depletion in humans was shown to elicit overlapping neurocognitive effects (i.e., impaired decision-making) with those observed in chronic METH users [45]. Thus, a peripheral TRY depletion may mediate the clinical phenotype of chronic METH use and represent a relevant target for treatment development. It should be noted that a previous study with experimental manipulation of dietary TRY failed to elicit pro-cognitive effects in MDMA users [46]. However, comparable investigations have not yet been conducted in METH users, in whom blood TRY levels appear to be affected. Such studies would be supported by the present findings and the existing literature.

### Chronic METH use is associated with serum 5-HT reduction

The reduction of 5-HT levels in METH users is in line with previous preclinical studies. TRY metabolism into 5-HT is mediated by the rate-limiting tryptophan hydroxylase (TPH) enzyme. Both MDMA and METH have been widely shown to inhibit brain TPH activity and thus reduce brain 5-HT availability both acutely and long-term [47–49]. However, substance effects on systemic TPH have received far less attention. This distinction is particularly relevant as peripheral 5-HT does not cross the BBB. Accordingly, body and brain 5-HT are considered as parts of two distinct systems, with different biological implications. In our sample, reduced blood 5-HT might be a direct consequence of lower TRY availability, which is supported by unchanged 5-HT/TRY ratios across samples and normal 5-HT levels in MDMA users. Nonetheless, a modulation of THP activity beyond the brain is also possible. The observation of reduced serum 5-HT levels gains particular relevance in view of emerging evidence on the role of 5-HT in brain-body interactions [17]. Peripheral 5-HT has been recognized as a modulator of autonomic signalling via vagus nerve stimulation [50]. Blood 5-HT reduction has been causally linked to fatigue and neurocognitive functioning in post-viral syndromes, also via altered afferent nerve stimulation [17]. Moreover, body 5-HT levels modulate innate and adaptive immune responses and shape gut-immune interactions [51–53]. Some limited evidence has linked 5-HT-dependent immune-system alterations to mental health conditions such as affective disorders, OCD and autism [54–56]. Accordingly, alterations of body 5-HT pathways might be involved in chronic METH use. Importantly, METH effects on 5-HT in our study were associated with urine positivity with strong effect sizes. Therefore, this effect is most likely related to recent METH use rather than a long-term alteration. Thus, the interaction between METH and 5-HT metabolism may be reversible through abstinence.

### Differential effects of MDMA and METH on the KYN pathway

While METH was associated with general activation of KYN metabolism but no alteration in the balance between the KYNA and OH-KYN branches, MDMA was associated with selective activation of the OH-KYN branch. Previous data on the effects of amphetamine derivates on KYN metabolites are very limited [24]. Consistent with our findings, Wang and colleagues previously reported increases in KYN/TRY and 3-HK/KYN ratios, as well as TRY depletion, but no changes in absolute KYN metabolite concentrations in METH users compared to controls [24]. In rats, MDMA has been shown to induce an acute and subacute increase of KYN metabolism through indoleamine 2,3-dioxygenase activation (converting TRY to KYN), without direct evidence for downstream effects on the OH-KYN branch [57]. Older rodent studies further suggest that amphetamine derivatives may modulate kynurenine aminotransferase activity (converting KYN to KYNA), while evidence for the kynurenine 3-monooxygenase (KMO, converting KYN to OH-KYN) is lacking [58]. Therefore, the activation of the OH-KYN branch in MDMA users is newly described and not clearly explained. Potential activation of the OH-KYN branch through KMO upregulation has been described for pro-inflammatory cytokines,reactive oxygen species, and high O2 concentrations [59, 60]. Notably, antidepressants (i.e., ketamine and selective serotonin reuptake inhibitors [SSRIs]) have been suggested to reduce (directly or indirectly) KMO activity [61, 62]. Thus, MDMA and METH appear to differ from antidepressants in their effects on KMO, although differences in study design between antidepressant studies and the current and Wang’s investigations prevent us from making conclusive statements in this regard. Looking at the implications, OH-KYN crosses the BBB and elicits glutamatergic modulation (NMDA-R agonism) through its conversion into quinolinic acid. Neurotoxic effects of quinolinic acid have been widely described in preclinical studies and also suggested in humans [63, 64]. Nonetheless, we did not observe consistent changes in the balance between the KYNA and OH-KYN branches across substances. Moreover, MDMA effects were limited to an increase in OH-KYN/TRY ratio without increasing absolute OH-KYN levels.

Overall, we demonstrated a different profile of KYN pathway alterations between METH and MDMA users, with relevant implications for their contrasting clinical presentation. On the contrary, no effects of MDMA and METH on IND metabolites were observed, thus suggesting a limited role of these gut-derived metabolites in the chronic effects of the substances.

### METH use is associated with stronger network-level changes

In our study, METH and MDMA showed different organization of TRY pathways at the network level. Network approaches are increasingly used in psychiatry, for both symptoms and biological measures, as they can reveal latent alterations not apparent from absolute values [65, 66]. To date, there is limited literature applying network analyses to TRY pathways, and our study provides initial evidence supporting their utility. In this exploratory analysis, stronger alterations were observed in the METH sample, with higher edge weights and disrupted overall network topology. Closeness centrality, reflecting node communication efficiency, was higher in MDMA, while eccentricity, reflecting distance to other nodes, was lower, both consistent with reduced pathway integrity in METH users. Given the sample size, these findings should be interpreted as global pathway-level effects rather than reliable changes at individual nodes. Nonetheless, the network results complement the metabolite-level analyses, highlighting differential TRY pathway reorganizations between METH and MDMA and supporting the use of network approaches in future studies of peripheral metabolic systems.

### Associations between metabolites, ratios, and psychopathology

Our study provides novel evidence on the contribution of peripheral TRY pathways in shaping the clinical symptoms related to chronic MDMA and METH use. We particularly found that depressive symptoms (i.e., the CESD score) are negatively associated with TRY and KYNA levels, while being negatively associated with general activation of the KYN pathway. These findings are largely consistent with the literature on blood TRY levels in affective disorders [67]. Experimental depletion of blood TRY has been particularly associated with depressive symptoms through down-regulation of brain 5-HT production [19]. While peripheral 5-HT depletion does not necessarily reflect reduced brain concentrations, a potential pathway associating reduced blood 5-HT with affective symptoms through autonomic nerve signalling has been proposed [17, 50]. General activation of the KYN pathway through induction of the indoleamine 2,3-dioxygenase (i.e., by subclinical pro-inflammatory mediators and chronic stress) was also associated with depression [67]. Brain KYNA elicits NMDA-R antagonism and neuro-protective effects, thus KYNA reduction is also consistent with higher risk for depressive symptoms [22]. However, blood KYNA poorly crosses the BBB and its impact on brain KYNA is therefore questionable. In this context, a systematic review comparing TRY pathways in CSF and blood reported moderate to strong CSF–blood correlations for KYN and OH-KYN, but inconsistent findings for KYNA and TRY [68]. Nonetheless, blood KYNA alterations might still reflect an alteration in the KYN pathway which might elicit indirect effects on brain NMDA-R modulation.

Psychotic symptoms (i.e., CAPE score) were associated with lower TRY and KYNA. These findings are contrary to the KYNA hypothesis of schizophrenia, which suggests that KYNA-dependent glutamatergic modulation shapes psychosis risk [69]. However, blood KYNA does not reflect brain KYNA levels and was found to have limited association with psychotic symptoms [70]. On the contrary, increased OH-KYN (which crosses the BBB) and OH-KYN pathway activation have been associated with psychotic symptoms in neuroleptic-naïve individuals with first-episode psychosis [71, 72]. The observed positive associations between psychotic symptoms, lower KYNA/OH-KYN ratios, and higher OH-KYN/TRY ratios are consistent with this pathway. Potential mechanisms include non-linear modulation of 5-HT and glutamatergic neurotransmission, as well as activation of neurotoxic pathways through higher quinolinic acid production. However, given the cross-sectional and transdiagnostic nature of the analysis, the observed associations remain correlational, and causal relationships cannot be inferred, underscoring the need for longitudinal and experimental studies to determine whether tryptophan pathway alterations contribute to psychopathology or reflect downstream consequences.

### Limitations

Our study bears some limitations. First, the cross-sectional nature of our investigation limits us in addressing the causal links between substance use and metabolite levels. In this context, the association between psychopathology scores and metabolite levels and ratios cannot disentangle effects of chronic substance use from pre-existing vulnerability. This is also limited by the intrinsic collinearity between psychiatric symptom scores and group membership, considering the higher severity in METH users. Future studies should include longitudinal data to better understand the dynamic course of this association and provide more robust evidence of dose-response relationships. Second, the lack of cerebrospinal fluid samples in the total sample and neuroimaging data in METH users do not allow us to assess potential associations between blood metabolites and brain functions. Nevertheless, peripheral alterations in these blood metabolites have been shown to modulate brain activity and behavior through both direct mechanisms, such as KYN and OH-KYN crossing the blood–brain barrier, and indirect mechanisms, including 5-HT effects on afferent nerves and the autonomic system, and TRY shaping protein and 5-HT synthesis via availability. Thus, blood alterations represent a distinct yet relevant body-first mechanism of chemical adaptation that diverges from brain-first effects but can still influence clinical outcomes. Third, chronic MDMA and METH use differ in use pattern and amount, thus affecting direct pharmacological comparisons. However, this reflects the difference in their real-world use and is therefore intrinsic in the naturalistic investigation of their contrasting clinical implications. While a full disentanglement of the relative contributions of dose, use pattern, and pharmacological profile is not possible, control analyses for acute use and dose–response investigations suggest that the observed effects are not solely driven by differences in cumulative dose exposure or use recency. Relatedly, the groups differed in other substance use (i.e., cannabis, cocaine, alcohol, and nicotine) as well as psychotropic medication exposure, which cannot be fully disentangled and may represent residual confounding. However, this concern has been mitigated by sensitivity analyses indicating limited contribution of these factors to the observed effects. Similarly, sex distribution was unbalanced in the user groups, which is also largely inevitable given that males are usually overrepresented in METH compared to MDMA user samples. Lastly, the moderate sample size might affect statistical power and significance detection at lower effect sizes. Furthermore, future studies should include a characterization of inflammatory markers and enzyme activity to better clarify potential pathways involved. Nonetheless, we provide a robust demonstration of the contrasting effects of MDMA and METH on TRY pathways with relevant clinical implications.

### Conclusions

The current investigation sheds light on the distinct alterations of peripheral TRY pathways in METH vs. MDMA users. We demonstrated substantial metabolite alterations in METH users (mostly driven by a reduction of TRY and 5-HT and an activation of the KYN pathway) and limited changes in MDMA users. We validated these findings using both case-control comparison of metabolite levels and an explorative network analysis approach. The metabolic changes showed relevant associations with psychopathology symptoms in the depression and psychosis domains, thus supporting the involvement of these pathways in the clinical presentation of chronic METH vs. MDMA use. Overall, our findings suggest that differences in clinical severity between METH and MDMA users may reflect not only central but also systemic metabolic alterations, pointing beyond a purely brain-focused view of psychoactive compound effects. Future studies should clarify the causal relationship between MDMA and METH use and changes in TRY-pathways and test experimental manipulations targeting these metabolites for treatment purposes.

## Data Availability

The data that support the findings of this study are available from the corresponding author upon reasonable request, subject to appropriate ethical and institutional approvals.

## Code availability

Analyses were conducted in Python using standard statistical libraries. No custom algorithms were developed; scripts used for data processing and analysis are available from the corresponding author.
